# *De Novo* Donor-Specific HLA Antibody Development and Peripheral CD4^+^CD25^high^ Cells in Kidney Transplant Recipients: A Place for Interaction?

**DOI:** 10.1155/2012/302539

**Published:** 2012-08-23

**Authors:** Josefina Alberu, Maria Inés Vargas-Rojas, Luis E. Morales-Buenrostro, Jose C. Crispin, Roxana Rodríguez-Romo, Norma O. Uribe-Uribe, Gabriel Carrasco, Diana Gómez-Martín, Jorge Alcocer-Varela

**Affiliations:** ^1^Department of Transplantation, National Institute of Medical Science and Nutrition Salvador Zubirán, Vasco de Quiroga 15, Colonia Sección XVI, DF, 14000 Mexico City, Mexico; ^2^Department of Immunology, National Institute of Medical Science and Nutrition Salvador Zubirán, Vasco de Quiroga 15, Colonia Sección XVI, DF, 14000 Mexico City, Mexico; ^3^Department of Pathology, National Institute of Medical Science and Nutrition Salvador Zubirán, Vasco de Quiroga 15, Colonia Sección XVI, DF, 14000 Mexico City, Mexico

## Abstract

The aim of this study was to determine whether the abundance of regulatory T cells (Tregs) (CD4^+^CD25^high^) affects the *de novo* development of anti-HLA donor-specific antibodies (DSAs) in kidney transplant recipients (KTRs). *Methods*. Unsensitized (PRA ≤ 10%, no DSA) adult primary KTRs who received a living (83%) or deceased (17%) KT in our Institution during 2004/2005 were included. DSA testing was performed monthly, and Tregs were quantified by flow cytometry every 3 months, during the 1st year after KT. All patients received triple drug immunosuppressive therapy (CNI + MMF or AZA + PDN); 83% received anti-CD25. *Results*. 53 KTRs were included; 32% developed DSA during the 1st year after KT. Significantly lower 7-year graft survival was observed in those who developed DSA. No difference was observed in Treg numbers up to 9 months after KT, between DSA positive and negative. However, at 12 months after KT, DSA-negative patients had significantly higher numbers of Treg. *Conclusions*. Early development of DSA was not associated to variations in Treg abundance. The differences in Treg numbers observed at the late time point may reflect better immune acceptance of the graft and may be associated to long-term effects. Additional inhibitory mechanisms participating earlier in DSA development after KT deserve to be sought.

## 1. Introduction

Effective immunosuppressive regimens have greatly improved the early survival of renal allografts. However, the rate of late allograft loss has remained relatively constant [1]. This is probably related to the fact that late allograft dysfunction not only results from immune-mediated damage, but also occurs as the consequence of a complex series of events that include arterial fibrointimal thickening, interstitial fibrosis, and tubular atrophy [2–4].

The presence of donor-specific antibodies (DSAs) directed against human leukocyte antigens (HLAs) has been associated in a growing number of reports to poor prognosis of renal allografts [5–7]. The association between anti-HLA antibodies and poor renal allograft evolution is explained by diverse alloantibody-mediated clinical syndromes, ranging from hyperacute rejection [8], early and late acute alloantibody-mediated rejection [9–11], and chronic humoral rejection [12]. 

In contrast to the well-known acute and devastating effects of preformed antibodies, *de novo* produced antibodies against an implanted graft do not cause immediate failure [7]. However, *de novo* antibodies may eventually cause chronic graft rejection [13, 14].

The presence of DSA implies that B-cells bearing a B cell receptor able to bind to donor HLA have effectively presented alloantigens through the indirect pathway and have received T-cell help [15]. However, presentation of alloantigens may result in T-cell activation or in the generation of a tolerogenic response. The factors that determine if a proinflammatory or a regulatory response will prevail in an individual patient are unknown [16]. With this scenario as a background, we were interested in studying whether the *de novo* development of DSA could be influenced by the number of peripheral regulatory T cells (Tregs) during the first year after transplantation in kidney transplant recipients. Therefore, the aims of this study were to document (i) the development of *de novo* DSA during the first year after kidney transplantation, (ii) the abundance of peripheral Tregs during the same period, (iii) the temporal relationship between peripheral Treg numbers and the *de novo* development of DSA, and (iiii) the function and survival of renal allografts in a group of patients who received a kidney transplant during 2004 and 2005 at the Instituto Nacional de Ciencias Médicas y Nutrición Salvador Zubirán and were followed during at least 5 years. 

## 2. Subjects and Methods

### 2.1. Patients and Sera Samples

We included in this prospective study all adult patients (>18 yrs) who received a primary kidney transplant from either a living or deceased donor in our institution during 2004 and 2005 and met the following criteria: current negative T-cell and B-cell AHG-CDC crossmatches; PRA ≤ 10%; absence of DSA class I and class II. During the first year after transplantation, monthly blood samples were drawn for DSA testing, and once every 3 months for Tregs quantification. Clinical data, gathered both at baseline and prospectively, included demography, cause of renal failure, type of renal replacement therapy, pretransplant blood transfusions, pregnancies, donor source, shared haplotypes for living related donors, or HLA mismatches for living unrelated and deceased donors, use of induction therapy, immunosuppressive schedule, biopsy-proven acute rejection episodes during the entire followup, graft function at 3, 12, and yearly (≥60 months posttransplant) thereafter until last followup, time and cause of graft loss or death. Institutional Review Board approval was obtained to conduct this trial, and all participant patients signed an informed consent.

### 2.2. Immunosuppressive Regimen 

Induction therapy with 2 mg/Kg (total dose) of Daclizumab was administered to all kidney transplant recipients (KTRs), except in 2 cases that had a 2-haplotype match. The immunosuppressive treatment included (a) Cyclosporine (target plasma levels 175–200 ng/mL during the first 3 months and ~150 ng/mL thereafter) or Tacrolimus (target plasma levels 8–12 ng/mL during the first 3 months and ~5 ng/mL thereafter); (b) an antiproliferative drug, either azathioprine (1.5–2 mg/Kg), or mycophenolate mofetil (2 g/day when combined with cyclosporine; 1.5 g/day with tacrolimus); (c) methylprednisolone 10 mg/Kg on transplant day, followed by daily boluses of 500 mg, 250 mg, and 125 mg, followed by prednisone starting on 100 mg on the 5th posttransplant day and gradually tapering down to 5 mg/day after 3 months. 

### 2.3. Donor-Specific Antibodies Assessment

KTRs and their donors were HLA typed before the transplant using LABTypeSSO (One Lambda) according to the manufacturer's instructions. All pre- and posttransplantation sera were tested for the presence of HLA class I and class II IgG antibodies using LABScreen Mixed according to the manufacturer's instructions (One Lambda, Inc., Canoga Park, CA). All sera positive for HLA antibodies (class I or II) were additionally tested for DSA with single antigen LABScreen beads (One Lambda Inc., Canoga Park, CA). Briefly, 20 *μ*L of serum samples were incubated with HLA class I-coated and HLA class II-coated microspheres, respectively, for 30 minutes in the dark under gentle agitation. The specimens were then washed five times before being incubated with anti-human IgG-conjugated phycoerythrin in the same conditions as in the first incubation. The Labscan 100 flow analyzer (Luminex, Austin, TX) was used for beads and data acquisition. Data were then analyzed with HLA Visual software (One Lambda). The cut-off level was defined as a baseline normalized >500 mean fluorescence intensity units (MFI). The presence of DSA was assigned by comparing the various HLA specificities proposed by the software analysis with the HLA typing of the donor for all the transplanted patients. 

### 2.4. Peripheral Tregs Quantification

**Figure 1:**
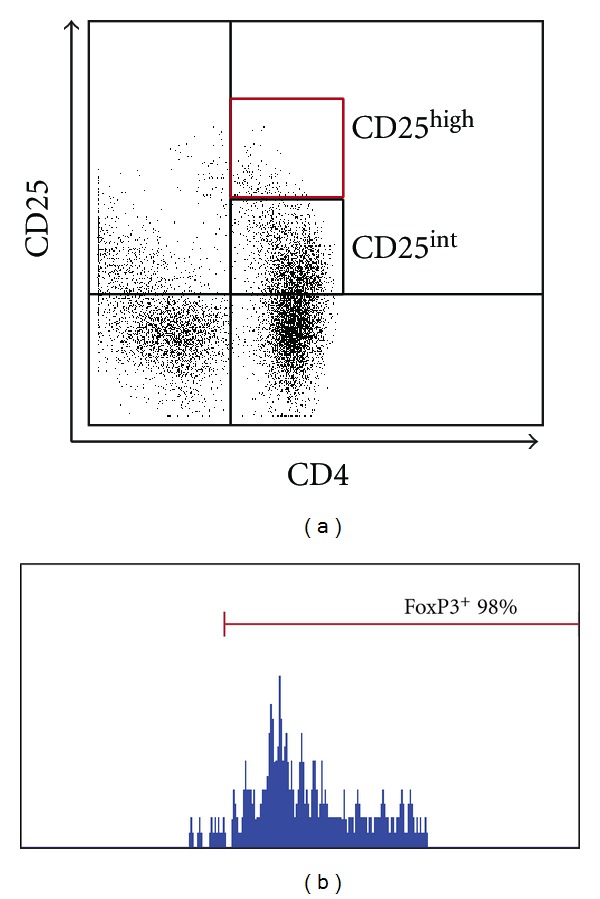
Regulatory T cells were defined as CD4^+^CD25^high^ T cells. (a) Cells were quantified using the gate indicated in red. A representative dot plot is shown. (b) Virtually all CD4^+^CD25^high^ cells express high levels of FoxP3.

Peripheral blood mononuclear cells (PBMCs) were obtained from patients and healthy donors by density-gradient centrifugation (Lymphoprep). PBMCs were stained with anti-CD25-PE (Clone M-A251, BD Pharmingen) and anti-CD4-PerCP (BD Pharmingen). In some experiments, cells were fixed and permeabilized with cytofix/cytoperm (BD Pharmingen) and intracellular staining (anti-FoxP3-FITC) was performed according to the instructions of the manufacturer. Data were collected on a FACScan flow cytometer (BD Biosciences). A CD25^high^ gate that included >95% FoxP3^+^ cells was defined in normal subjects (Figure 1) [17]. This gate, that included ~2% of CD4^+^ T cells, was used for the quantification of CD25^high^ cells in all patients.

### 2.5. Acute Rejection Definition and Treatment

Acute graft dysfunction was defined as an unexplained increase ≥ 25% in serum creatinine (SCr). In all cases, graft biopsy was performed and urinary obstruction and/or infection were ruled out. Acute rejection was defined according to Banff ′97 and the ′03 update working classification of renal allograft pathology [18, 19]. Treatment for acute rejection consisted of 3 days of high dose I.V. methylprednisolone (12 mg/Kg/day). Acute cellular (ACR) steroid-resistance rejections were treated with thymoglobulin. Acute antibody mediated rejection (AMR) episodes were treated with 3 sessions of plasmapheresis (PP), followed each by 100 mg/Kg body weight IVIG, and Rituximab.

### 2.6. Graft Function at Followup

Data required for glomerular filtration rate estimation (eGFR) according to the Modification of Diet in Renal Disease (MDRD) formula, at 1, 12 months, and last followup after KT was prospectively gathered from medical records.

### 2.7. Statistical Analyses

For continuous variables, differences between two groups were evaluated by independent sample Student's *t* or Mann-Whitney *U* tests according to data distribution (normal or abnormal, resp.). We used Chi-square test for categorical variables. The Fisher exact test was used when expected values were under 5. The association between HLA mismatches and DSA status was evaluated with Chi-Square for trend. To compare all measurements of Treg during 1 year, we used Kruskal-Wallis test. Graft survival was analyzed using the Kaplan-Meier method with log rank test for comparison of survival curves. A *P* < 0.05 value was considered statistically significant.

## 3. Results

### 3.1. Study Population

 Seventy-six first kidney transplants were performed in our center from January 2004 to December 2005. Fourteen out of 76 KTRs had a pretransplant PRA > 10% and therefore were not included. Sixty-two patients were enrolled in the study, but 9 were not considered in the final analysis. Reasons for excluding these patients were as follows: death during the first 12 months (*n* = 2) and followup in another institution (*n* = 7) during the 1st year after transplant. Therefore, the total number of KTRs included in this analysis was 53.

Baseline characteristics of the patients are shown in Table 1. No differences were observed between patients who remained DSA negative and those who developed DSA during the first year after transplant, regarding demography, ESRD cause, antecedent of sensitizing events, donor type, HLA mismatches, or immunosuppressive treatment. According to the inclusion criteria, all patients should have had a pretransplant PRA ≤ 10% and absence of DSA. PRA was 0% for 46 (86.8%) patients; 7 (13.2%) patients had a PRA class I between 2 and 7%, and only 1 of them had a PRA class II of 3%.

### 3.2. DSA Development during the First 12 Months after Transplantation

**Figure 2:**
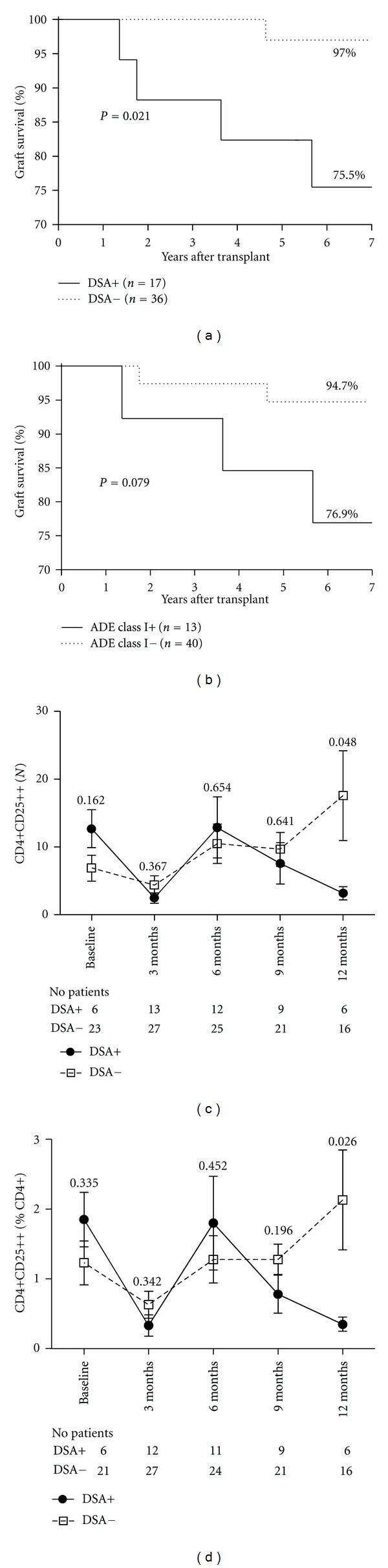
(a) Graft survival according to DSA development during the first year post-KT; DSA+ve versus DSA−ve, *P* = 0.021. (b) Graft survival according to class I DSA development during the first year post-KT, *P* = 0.079. Treg numbers (c) and percentages (d) at different time points during the first year post-KT in patients who developed DSA and those who remained DSA−ve. At 12 months, the difference in Treg numbers and percentages was significantly different between DSA+ve versus DSA−ve patients, *P* = 0.048, and *P* = 0.026, respectively.

Seventeen patients (32%) developed DSA during the first year after KT. Class I DSA, class II DSA, or both were detected in 11, 4, and 2 patients, respectively. As shown in Figure 2(a), development of DSA during the first year after KT was significantly associated to lower graft survival (*P* = 0.021). Graft survival was also lower in patients who developed class I DSA (Figure 2(b)); however, the difference did not reach statistical significance (*P* = 0.079). No difference was associated to development of class II DSA (*P* = 0.494) (data not shown).

### 3.3. Quantitative Profile of Regulatory T Cells (CD4^+^CD25^high^) during the First 12 Months after Transplantation and Its Relationship to DSA Development

Tregs were considered as absolute number (cells/*υ*L) and as percentage of CD4^+^ T cells. The median (interquartile range) absolute number detected at baseline and during months 3, 6, 9, and 12 were 6.45 (1.7–10.9), 1.49 (0.2–5.0), 5.98 (2.3–11.9), 5.51 (3.9–8.6), 4.81 (2.1–14.6), respectively (*P* < 0.001); these correspond to 1.20% (0.4–1.7), 0.16% (0.02–0.52), 0.97% (0.34–1.44), 0.80% (0.41–1.69), and 0.68% (0.29–1.84), respectively (*P* < 0.001). There was a decrease in Treg number at 3 months. This was probably associated to the effect of anti-CD25 induction therapy. At month 6, the abundance of peripheral blood Tregs tended to increase and remained similar to the baseline number up to the end of the followup (12 months).

Between month 6 and the end of the first year, the numbers of peripheral blood Tregs differed between the patients who developed DSA and those who did not. A progressive decrease in Treg numbers was observed in patients who developed DSA. In contrast, an increase in this population was observed in patients who remained DSA negative (Figures 2(c) and 2(d)). Importantly, the decrease in Treg numbers was specifically associated with development of class I DSA (*P* = 0.023). 

### 3.4. Acute Rejection Episodes

The number of biopsy-proven acute rejection episodes that occurred during the entire followup was 11.  Table 2 describes the patients who developed acute rejection episodes, the number of events confirmed in these patients, and the type of acute rejection documented, according to DSA status. There was a significant difference in the number of patients who experienced acute rejection episodes in the DSA-positive group compared to those in the DSA-negative group (*P* = 0.01).

### 3.5. Graft Loss and Patient Death

Five grafts were lost during followup. The causes of graft loss in these patients corresponded to biopsy-confirmed chronic antibody-mediated rejection (1), grade III chronic allograft nephropathy (1), grade III acute and chronic antibody mediated rejection plus thrombotic microangiopathy (1), acute cellular rejection 1B superimposed to a chronic vascular rejection (1), and acute and chronic cellular mediated rejection (1). 

It is worth mentioning that 4 out of these 5 patients belonged to the group that developed DSA during the first 12 months after transplantation (Figure 2(a)). Patient survival was 100%.

### 3.6. Graft Function

One of the most pursued aspects of the surveillance of these patients was the evolution of renal function for at least 5 years after transplantation. Overall, graft function assessed through SCr and MDRD eGFR shows that regardless DSA status during the first year, there was an increase in SCr as time elapsed. Albeit not significant, deterioration of graft function was more evident in the DSA-positive group according to this parameter (Table 3). When we analyzed delta eGFR (last SCr obtained in 2010—1 month after transplantation), the DSA-negative group showed a mean positive slope of 13.14 mL/min, while a mean slope of −0.41 mL/min was observed in the DSA-positive group. This analysis only included patients with graft function. 

## 4. Discussion

We studied prospectively a cohort of DSA-negative renal transplant patients and quantified the numbers of peripheral blood regulatory T cells (defined as CD4^+^CD25^high^) at different time points during the first year. We found that, as expected, Treg numbers dropped following administration of anti-CD25, but their numbers recovered 6 months after transplantation. Moreover, we observed that, in a subset of the patients, Treg numbers remained stable and tended to increase towards the end of the first year. These patients remained DSA negative, and their renal allograft had a better outcome. We also detected a group of patients whose Treg numbers decreased in the second semester after transplantation. DSA development was associated with this phenomenon. 

Currently, one of the most challenging aspects in the field of kidney transplantation is the unmet need to translate the fantastic improvements achieved in first-year graft survival—exceeding 90%—into long-term graft survival [1]. Antibody-mediated injury is increasingly recognized as a factor implicated in long-term graft attrition [20, 21]. Important contributions have identified the clinical significance of DSA before the transplant [22] and when their production follows the procedure [14].

One of the purposes of our study was to evaluate the significance of *de novo* DSA developed during the first-year after kidney transplantation. A significantly higher number of patients in the DSA-positive group developed acute rejection episodes compared to the patients that remained DSA negative. Also, long-term graft survival was reduced significantly in the former group of patients. Three out of four biopsies performed in DSA-positive patients who lost the graft revealed acute and/or chronic AMRs combined with findings of T-cell-mediated rejection. In the other patient, a T-cell-mediated rejection dominated the picture. In general, all these rejections corresponded to late AMR that have been associated to *de novo* DSA [23]. Also, it has been suggested that late AMR episodes pose a worse long-term prognoses compared to early AMR episodes. In this study, *de novo* DSA represented a biomarker for graft loss, as has been previously suggested [24]. Most probably DSA development detected during the posttransplant evolution translates a heightened immune response, which, associated to nonadherence or to other factors, provides an opportunity for intervention to prolong graft survival. 

The impact that *de novo* DSA conveys to graft survival has been demonstrated previously [14, 25]. Nickerson et al. [26] described 3 presentation patterns related to *de novo* DSA. The histological findings in the grafts of the patients included in our study where acute and/or chronic AMRs are combined with findings of T-cell-mediated rejection suggest that nonadherence to treatment could have been a participant factor [23, 25].

One of the aims of our study was to explore the behavior of the number of Tregs during the first year after transplantation in nonsensitized kidney transplant recipients, and the temporal relationship between Treg numbers and *de novo* DSA development. Naturally occurring CD4^+^ Tregs constitutively express high levels of the IL-2 receptor alpha chain (CD25), are generated in the thymus, and display a powerful suppressive capacity [27]. Tregs are able to regulate the activity of several types of immune cells including effector T cells and B cells [28]. 

We were able to detect a drop in the number of CD4^+^CD25^high^ T cells at month 3 after transplant. This finding is in agreement to the data published by Segundo et al. [29] and most probably represents the effect of the anti-CD25 monoclonal antibody administered as induction therapy. Previous studies have shown that the majority of Tregs in humans express high levels of CD25 [30], and Bluestone et al. showed that basiliximab caused a transient loss of both FoxP3^+^ and FoxP3^−^ CD25^+^ T cells [31]. In the patients studied in our series, the number of CD4^+^CD25^high^ recovered by month 6 and remained stable during the first year after transplant. 

No differences in Treg numbers were evident during the first 6 months between the patients who developed or not DSA (Figures 2(c) and 2(d)). However, a progressive increase in Treg number apparent at the 9th month was observed in the group of patients who remained DSA negative. In sharp contrast, Treg numbers dropped in the group that eventually developed DSA. The difference in CD4^+^CD25^high^ T cells between DSA positive/negative groups was significant at month 12. It is interesting to note that more DSA-positive patients received induction therapy with anti-CD25 compared to DSA-negative patients (94% versus 72%). Even though the difference did not reach statistical significance, the trend suggests that administration of anti-CD25 therapy might in some cases cause a prolonged decrease in Treg numbers. 

Immunophenotyping was not carried out in the performed biopsies, and protocol biopsies were not performed in patients whose renal function remained stable and were DSA negative. This would have provided us with valuable material to compare the cellular infiltrate in both conditions (rejection versus stable grafts). This represents a weakness of the study. Another weakness of the study is that Tregs were defined as CD25^high^. Since CD25 is a cell activation marker, CD25^+^ cells may represent activated T cells. Other markers, in particular FoxP3, are more specific for Tregs. Nevertheless, we believe that we did not overestimate the number of Tregs because we gated in CD25^high^ cells that were virtually all FoxP3^+^ (Figure 1) and because we found that a lower number of Treg was associated with more inflammation. If we were including activated T cells, the bias would have been in the inverse direction. 

Summing up, we have presented data that suggests that CD4^+^CD25^high^ cells display a protective role against DSA development during the 1st year after KT. Additional inhibitory mechanisms participating earlier in DSA development after KT deserve to be investigated. 

## Figures and Tables

**Table 1:** Patient characteristics.

	All *N* = 53 (%)	DSA negative *N* = 36 (%)	DSA positive *N* = 17 (%)	*P*
Recipient female	30 (56.6)	20 (55.6)	10 (58.8)	1.000
Donor female	30 (56.6)	21 (58.3)	9 (52.9)	0.942
Recipient age, mean ± SD	32.3 ± 12.4	31.1 ± 12.2	34.8 ± 12.7	0.317
Donor age, mean ± SD	38.5 ± 10.9	37.9 ± 10.3	39.8 ± 12.1	0.558
ESRD etiology				
Diabetes	6 (11.3)	4 (11.1)	2 (11.8)	0.693
Lupus	3 (5.7)	3 (8.3)	0 (0)	0.556
Glomerulonephritis	3 (5.7)	3 (8.3)	0 (0)	0.556
Hypertension	2 (3.7)	2 (5.6)	0 (0)	0.827
Other	3 (5.7)	1 (2.8)	2 (11.8)	0.494
Unknown	36 (67.9)	23 (63.9)	13 (76.4)	0.548
Sensitizing events				
Blood transfusions	35 (66.0)	23 (63.9)	12 (70.6)	0.865
Pregnancies (*N* = 30)	11 (36.7)	5 (25.0)	6 (60.0)	0.108
Donor type				
Living donor	44 (83)	29 (80.6)	15 (88.2)	0.701
Deceased donor	9 (17)	7 (19.4)	2 (11.8)	
HLA mismatches				0.040^∗^
0	6 (11.3)	6 (16.7)	0 (0)	0.186
1	5 (9.5)	4 (11.1)	1 (5.9)	0.917
2	6 (11.3)	5 (13.9)	1 (5.9)	0.693
3	15 (28.3)	9 (25)	6 (35.3)	0.652
4	2 (3.8)	2 (5.6)	0 (0)	0.827
5	13 (24.5)	6 (16.7)	7 (41.1)	0.111
6	6 (11.3)	4 (11.1)	2 (11.8)	0.693
Immunosuppression				
Daclizumab	42 (79.2)	26 (72.2)	16 (94.1)	0.082
CsA+Aza+Pdn	9 (17.0)	6 (16.7)	3 (17.6)	0.762
CsA+MMF+Pdn	3 (5.7)	3 (8.3)	0 (0)	0.556
Tac+Aza+Pdn	18 (34.0)	13 (36.1)	5 (29.4)	0.865
Tac+MMF+Pdn	22 (41.5)	14 (38.9)	8 (47.1)	0.791
Other	1 (1.8)	0 (0)	1 (5.9)	0.698

All the patients had a PRA < 10% at transplantation and absence of donor-specific antibodies (DSAs). *P value by Chi square for trend.

**Table 2:** Acute rejection episodes.

	DSA− *N* = 36	DSA+ *N* = 17
Number (%) of patients with acute rejection episodes	2 (5.6)	6 (35.3)^∗^
Number of acute rejection episodes	3	9
Banff 97 grade and the 03 update working classification		
Acute/active cellular rejection		
Mild acute (IA)	1	1
Mild acute (IB)	0	3
Moderate acute (IIA)	1	1
Moderate acute (IIB)	0	0
Severe acute (III)	0	1
Antibody-mediated rejection		
I	0	0
II	1	2
III	0	1

*P = 0.010 (Fisher exact test).

**Table 3:** Graft function according to DSA development (1st year after KT).

	All *N* = 53 (%)	DSA negative *N* = 36 (%)	DSA positive *N* = 17 (%)	*P*
Graft function at 1 month				
SCr (mg/dL)	1.22 ± 0.33	1.27 ± 0.33	1.11 ± 0.30	0.096
eGFR (by MDRD, mL/min)	68.93 ± 27.18	65.37 ± 23.55	76.48 ± 33.15	0.167
Graft function at 12 months				
SCr (mg/dL)	1.23 ± 0.40	1.20 ± 0.32	1.29 ± 0.53	0.481
eGFR (by MDRD, mL/min)	65.69 ± 20.04	66.27 ± 19.53	64.44 ± 21.64	0.759
Deltas (12 months versus 1 month)				
SCr (mg/dL)	.0002 ± 0.39	−0.07 ± 0.36	0.15 ± 0.43	0.053
eGFR (by MDRD, mL/min)	−2.35 ± 21.65	0.62 ± 19.25	−8.66 ± 25.51	0.759
Graft function in 2010				
SCr (mg/dL)	1.95 ± 2.56	1.54 ± 1.91	2.79 ± 3.46	0.181
eGFR (by MDRD, mL/min)	74.41 ± 29.92	79.45 ± 26.96	64.03 ± 33.75	0.081
Deltas (2010 versus 1 month)				
SCr (mg/dL)	0.72 ± 2.59	0.26 ± 1.99	1.67 ± 3.40	0.129
eGFR (by MDRD, mL/min)	8.71 ± 27.59	13.14 ± 25.47	−0.41 ± 30.29	0.097
